# The Effectiveness of Semaglutide on a Composite Endpoint of Glycemic Control and Weight Reduction and Its Effect on Lipid Profile Among Obese Type 2 Diabetes Patients

**DOI:** 10.3390/medicina61081393

**Published:** 2025-07-31

**Authors:** Sumaiah J. Alarfaj

**Affiliations:** Department of Pharmacy Practice, College of Pharmacy, Princess Nourah bint Abdulrahman University, P.O. Box 84428, Riyadh 11671, Saudi Arabia; sjalarfaj@pnu.edu.sa

**Keywords:** diabetes mellitus, type 2, glucagon-like peptide-1 receptor agonist, semaglutide, effectiveness, weight reduction, A1C, composite, lipid profile, Saudi

## Abstract

*Background and Objectives:* Obesity and type 2 diabetes (T2D) are closely linked and associated with a higher risk of complications. This study aims to evaluate the effectiveness of once-weekly semaglutide in achieving a composite endpoint of A1C and weight reduction. *Materials and Methods:* This retrospective cohort study assessed the effectiveness of semaglutide in obese patients with T2D at a tertiary care hospital in Saudi Arabia. This study included patients who received semaglutide treatment for 12 months, and the endpoint was reducing A1C by ≥ 1% and body weight by ≥ 5% after 12 months of starting semaglutide. Secondary endpoints include predictors of achieving the composite endpoint and the effect on the lipid profile. *Results:* The present study enrolled 459 participants, with dyslipidemia and hypertension being the most common comorbidities. After 12 months of treatment with semaglutide, 42% of the patients achieved the composite endpoint. Semaglutide significantly reduced weight, BMI, A1C, FBG, total cholesterol, LDL, and triglycerides. The subgroup analysis showed that patients who achieved the composite endpoint were younger and had significantly lower use of insulin. Females in the study had significantly higher BMI, A1C, and HDL levels and lower levels of triglycerides compared to males. Multivariate analysis revealed that baseline BMI (aOR = 0.953; 95% CI: 0.915 to 0.992; *p* = 0.02), baseline A1C (aOR = 1.213; 95% CI: 1.062 to 1.385; *p* = 0.004), and receiving insulin (aOR = 0.02; 95% CI: 0.001 to 0.343; *p* = 0.007) were significant predictors of composite endpoint achievement. *Conclusions:* Semaglutide is a valuable option for the treatment of obese patients with T2D. This study found that semaglutide is effective in reducing weight and A1C and improving the lipid profile. The predictors of achievement of the composite endpoint were lower baseline BMI, higher baseline A1C, and insulin non-use.

## 1. Introduction

Obesity and type 2 diabetes mellitus (T2D) are quite interrelated, with obesity being the most important risk factor for the development of T2D [1]. Furthermore, the presence of obesity in T2D patients makes it more challenging to achieve glycemic control due to exacerbated insulin resistance [1,2]. The coexistence of obesity and type 2 diabetes is associated with a higher risk of complications, including cardiovascular disease, renal impairment, neuropathy, and retinopathy [3]. Despite efforts to promote a healthy lifestyle and diet, the prevalence of obesity and T2D continues to rise globally and in Saudi Arabia, leading to substantial health-related consequences and burdens on the healthcare system [4,5]. Likewise, nearly half of patients diagnosed with T2D continue to demonstrate suboptimal glycemic control, despite the availability of a wide range of antidiabetic medications and emerging therapeutic options. This highlights the continued necessity for research in the field, as numerous interventions, including medications and surgical procedures, have been proposed to manage obesity and T2D but are still underutilized [6].

The optimal management of diabetes in obese patients requires a comprehensive approach that ensures glycemic control and maintaining acceptable body weight to minimize the risk of complications and cardiovascular disease. Current guidelines recommend that healthcare providers should consider not only the glucose-lowering effects of prescribed medications, but also their potential impact on body weight [7]. An integrated multidisciplinary approach with an individualized treatment strategy is recommended for this population of patients [7,8].

Semaglutide is a glucagon-like peptide-1 receptor agonist (GLP1-RA) with 94% molecular similarity to human GLP1-RA and a half-life of almost one week [9]. It has been approved for the treatment of T2D and obesity [9]. GLP1-RAs exert their effect by stimulating insulin secretion, suppressing glucagon secretion, and promoting satiety. Multiple clinical trials have demonstrated the efficacy of semaglutide in reducing both weight and hemoglobin A1C (A1C) levels, improving metabolic profiles, and providing cardiovascular benefits by reducing the risk of major adverse cardiovascular events in patients with T2D and established cardiovascular disease [10,11]. Few side effects were associated with semaglutide, most commonly gastrointestinal side effects like nausea, vomiting, and constipation. Other less frequent side effects include pancreatitis, acute kidney injury, and diabetic retinopathy [12]. Additionally, the once-weekly injection regimen of semaglutide has been shown to improve treatment flexibility and patient adherence [13]. Furthermore, semaglutide has been associated with a favorable safety profile, satisfactory tolerability, lower risk of hypoglycemia, and fewer adverse events compared to other antidiabetic medications. Therefore, it is an ideal therapeutic option for patients who require simultaneous glycemic control and weight loss [10].

Composite endpoints are becoming more common as primary or secondary outcomes to tackle multiple desired outcomes simultaneously in clinical trials, and diabetes is not an exception [14]. Due to the high prevalence of obesity among patients with type 2 diabetes and the negative impact of obesity on glycemic control, it is important to consider the favorable effects of semaglutide on both diabetes control and weight reduction. Therefore, a composite endpoint that evaluates both A1C and weight reduction would provide a clinically meaningful assessment of treatment efficacy.

Despite the proven efficacy and safety of semaglutide, some studies have demonstrated considerable individual variations in the magnitude of the therapeutic response in terms of A1C and weight reduction [15]. It has been demonstrated that a weight loss of 5% and an A1C decrease of >1% in individuals with T2D are significant markers of a clinically meaningful response to treatment and have been shown to lower the risk of diabetes morbidity [16]. Additionally, there is a paucity of data evaluating the effectiveness of semaglutide in achieving a combined reduction in weight and A1C. Therefore, this study aims to assess the effectiveness of once-weekly semaglutide injections in achieving a composite endpoint of A1C reduction of ≥1% and ≥5% weight loss in Saudi patients.

## 2. Methods

### 2.1. Study Design, Setting, and Population

This single-center, retrospective, observational cohort study included all obese patients with T2D seen at Security Forces Hospital between 1 January and 31 December 2021. Data for this project were obtained by re-utilizing collected de-identified data from the researcher’s previously published project [17]. Security Forces Hospital, located in Riyadh, Saudi Arabia, is a tertiary care hospital with a 532-bed capacity and a diabetic care center. Patients were followed in the diabetes center and were all offered standard clinical practice for lifestyle modification recommendations. The dose started at 0.25 mg and was gradually increased to 1 mg according to the treatment protocol, patient response, and tolerability.

### 2.2. Sample Size Calculation

Based on previous studies, semaglutide has shown composite endpoint achievement rates ranging from 25% to 38% with a 0.5 mg dose and 38% to 59% with a 1 mg dose, compared to 2% to 23% for comparators [18]. We assumed a response rate of 42% (median of achievement in the semaglutide group). Using Cochran’s formula with a 95% confidence interval and a 5% margin of error, we calculated that a sample size of 361 was required to acquire a representative sample that achieve the composite endpoint.

**Figure 1:**
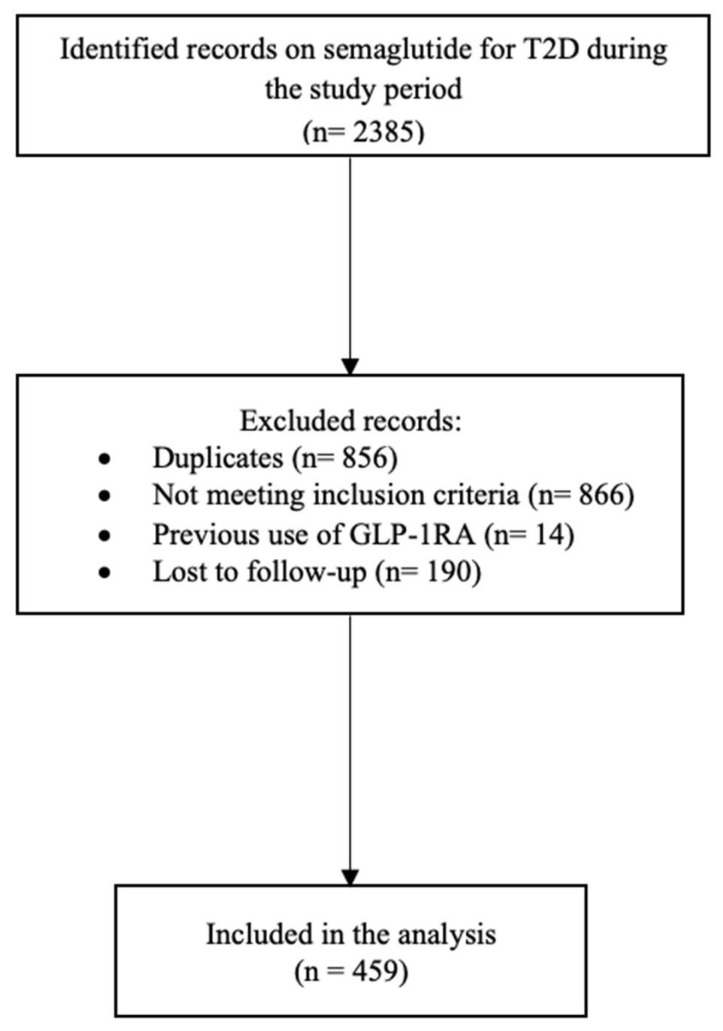
Enrollment process diagram.

We employed a convenience sampling technique and included all patients who met the inclusion criteria in our final analysis (459 patients). A detailed description of the inclusion diagram is depicted in Figure 1.

A post hoc sample size analysis revealed that our sample size, along with the observed effect size (odds ratio for composite endpoint achievement = 5.31), achieved a statistical power of more than 99% at an alpha level of 0.05.

### 2.3. Data Collection

During the recruitment period, data collection included all patients with T2D who were prescribed semaglutide injection and met the following criteria: age greater than 18 years, body mass index (BMI) greater than 30 kg/m^2^, A1C greater than 8%, and continued semaglutide treatment for at least 12 months. Patients who did not meet the inclusion criteria, had incomplete follow-up data, or had received GLP1-RA within the six months prior to the start of the study were excluded from the analysis.

### 2.4. Study Endpoints

We set a primary composite endpoint of reducing A1C by at least 1% from baseline and decreasing body weight by at least 5% after 12 months of starting semaglutide. Additionally, as secondary endpoints, we examined changes in FBG, BMI, and lipid profile at the study time points and investigated factors associated with achievement of the composite endpoint.

### 2.5. Ethical Considerations

The research committee of Princess Nourah bint Abdulrahman University (IRB log Number: 25-0464) approved this study in June 2025. Due to the retrospective nature of the research and the absence of personal identifiers in the data, informed consent was not acquired. Collected data were kept secure and accessible only to authorized study personnel throughout all stages of the study. The Declaration of Helsinki’s guiding principles were followed in the conduct of this investigation.

### 2.6. Statistical Analysis

Statistical analyses were performed using IBM SPSS software version 28. Continuous variables were checked for normality and presented as mean ± standard deviation. Categorical variables are shown as frequencies and percentages. Continuous variables were compared using two-sample *t*-test or paired *t*-test, while categorical variables were compared using Chi-squared or Fisher’s exact tests, as appropriate. Pearson’s correlation analysis was used to evaluate the correlations between study variables and General Linear Model Repeated Measures ANOVA to study longitudinal changes among different study time points. To determine the predictors, we conducted univariate and multivariable binary logistic regression, with achieving the composite endpoint of A1C reduction ≥ 1% and weight loss ≥ 5% as the dependent variable. To adjust for potential confounding, we included age, gender, BMI, and diabetes duration as covariates in addition to all variables from univariate analysis with *p* < 0.2. The interaction was examined by stratification and, if present, by the inclusion of an interaction term in the model to test for statistical significance. The goodness of fit of the model was assessed using the likelihood ratio test. All reported *p*-values are two-sided, and *p*-values < 0.05 were considered statistically significant.

## 3. Results

### 3.1. Baseline Characteristics

The current study included a total of 459 participants, with 213 (46.4%) being male and 246 (53.6%) being female. The average age of the participants was 52.7 ± 8.5 years, and the average duration of diabetes was 14.2 ± 7.8 years. The most common comorbidities observed were dyslipidemia in 417 (90.8%) patients, followed by hypertension in 247 (53.8%) patients. Almost all patients were on metformin, with 240 (52.3%) on insulin therapy and 124 (27%) on sulfonylurea.

At six months, 199 (43.4%) participants received a semaglutide dose of 0.5 mg, while 259 (56.4%) received a dose of 1 mg. At 12 months, 106 (23.1%) participants received a dose of 0.5 mg, while 350 (76.3%) received a dose of 1 mg (Table 1). Around 79% of patients remained at the same dose at 6 and 12 months. Out of the included patients, only four patients discontinued treatment with semaglutide during the follow-up period. Three were due to GI upsets, and one patient had no documented reason.

### 3.2. Composite Endpoint

A total of 193 patients (42%) achieved the composite endpoint, while 266 (58%) did not. In the comparison of baseline characteristics between the group that achieved the composite endpoint and the group that did not, we found that the group of patients who achieved the composite endpoint was younger. Additionally, the use of insulin was significantly lower in the group that achieved the endpoint, with 80 (41.5%) patients using insulin, compared to 160 (60.2%) in the group that did not achieve the endpoint (*p* < 0.001). The number of patients who received the 1 mg dose was slightly higher in the group that achieved the composite endpoint at 12 months, compared to the number of patients who received the 0.5 mg dose; nevertheless, there was no statistically significant difference in the rate of endpoint achievement between the two doses. All other baseline characteristics were comparable (Table 1).

### 3.3. Subgroup Analysis

Regarding sex/gender difference, we found that females in the study were significantly older than males and had a higher BMI, with values of 37.8 ± 5.7 for females and 34.7 ± 5.3 for males (*p* < 0.001). Females also had significantly higher levels of A1C and HDL and lower levels of triglycerides compared to males. At 6 months, the use of the 1 mg dose of semaglutide was lower in females compared to males, but this difference was not significant at 12 months (Table 2). Nonetheless, the outcome of achieving the composite endpoint was not different between males and females (87 (45.1%) vs. 106 (54.9%), *p*-value = 0.637).

### 3.4. Efficacy Outcomes and Metabolic Profile Changes

Table 3 provides a summary of the longitudinal changes in body weight and laboratory tests throughout the study time points, including data for the total patient population and subgroups of patients who did and did not achieve the composite endpoint. In the group that achieved the endpoint at 12 months, we found that 53 (27.5%) of them had already reached the endpoint at six months, compared to only 3 (1.1%) in the group that did not ultimately achieve the endpoint (*p* < 0.001).

**Figure 2:**
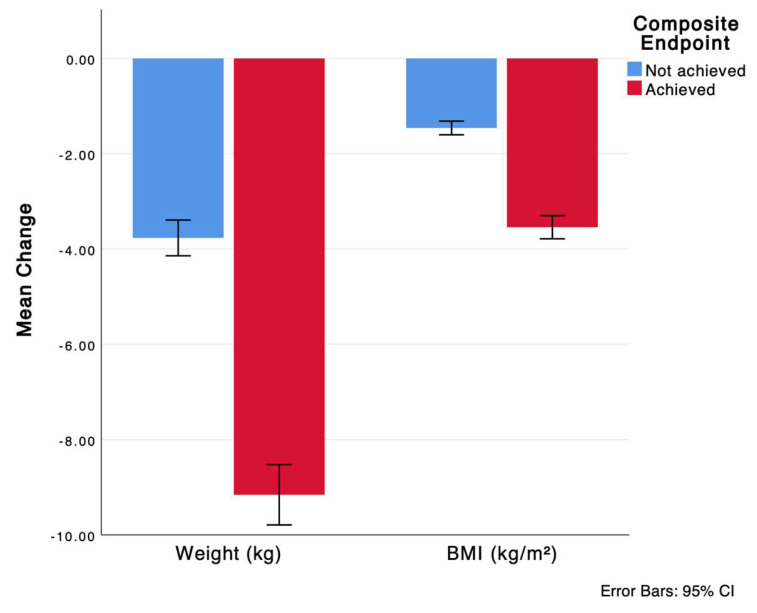
Change in weight and BMI 12 months from baseline by composite endpoint achievement. Refer to Table 3 for the numbers and *p*-values.

**Figure 3:**
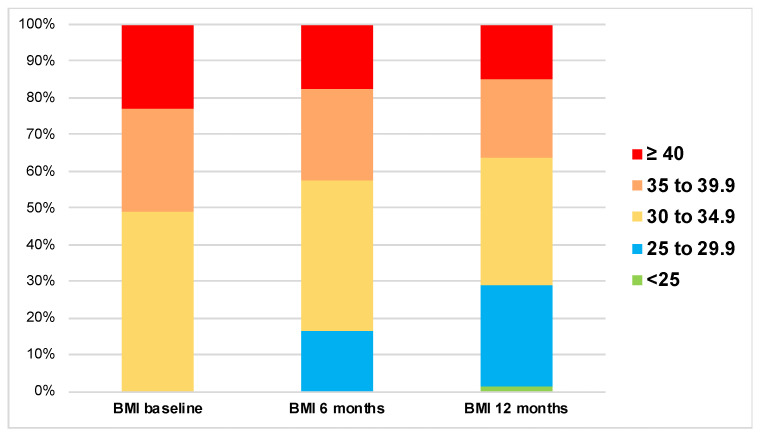
Change in the distribution of BMI categories at the study time points. *p* value < 0.001 obtained from related-samples Friedman’s two-way analysis of variance by ranks test.

Baseline weight, but not BMI, was significantly lower in the group that achieved the composite endpoint compared to the group that did not achieve the endpoint. However, both weight and BMI were significantly lower in the group that achieved the endpoint at 12 months (Table 3), with a higher change from baseline in the achieved group (Figure 2). Moreover, Figure 3 depicts the distribution of BMI categories at the different study time points.

**Figure 4:**
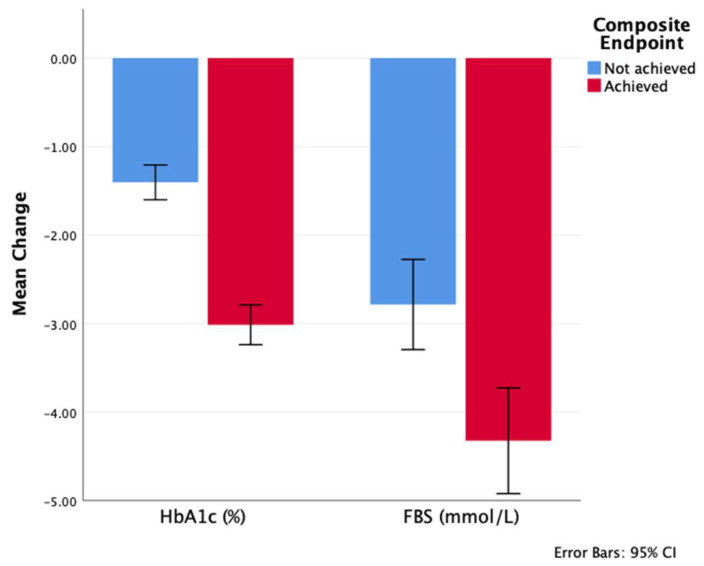
Change in FBG and A1C 12 months from baseline by composite endpoint achievement. Refer to Table 3 for the numbers and *p*-values.

**Figure 5:**
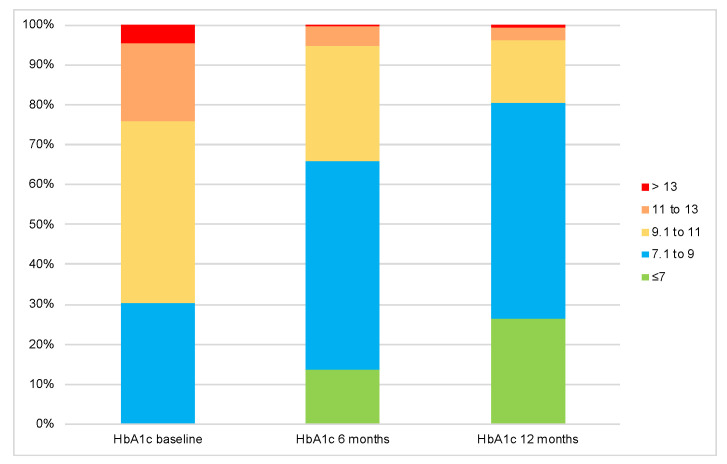
Change in the distribution of A1C categories at the study time points. *p* value < 0.001 obtained from related-samples Friedman’s two-way analysis of variance by ranks test.

Conversely, baseline A1C was significantly higher in the group that achieved the composite endpoint, with comparable fasting blood glucose (FBG) levels between the two groups. However, both A1C and FBG were found to be significantly lower at the end of the study in the group that achieved the endpoint (Table 3), with a higher change from baseline (Figure 4). Moreover, Figure 5 depicts the decreasing trend in A1C throughout the study time points.

In the entire study cohort, after 12 months of treatment with semaglutide, total cholesterol was significantly decreased from baseline by −0.21 mmol/L (95% CI: −0.31 to −0.11), LDL was decreased by −0.17 mmol/L (95% CI: −0.26 to −0.08), and triglyceride was decreased by −0.22 mmol/L (95% CI: −0.31 to −0.14), while the change in HDL was not significant (0 mmol/L; 95% CI: −0.02 to 0.02).

Across the three study time points, total cholesterol, LDL, and triglyceride levels were comparable between the group that achieved the composite endpoint and the group that did not. However, HDL levels were significantly higher at 12 months in the group that achieved the endpoint.

**Figure 6:**
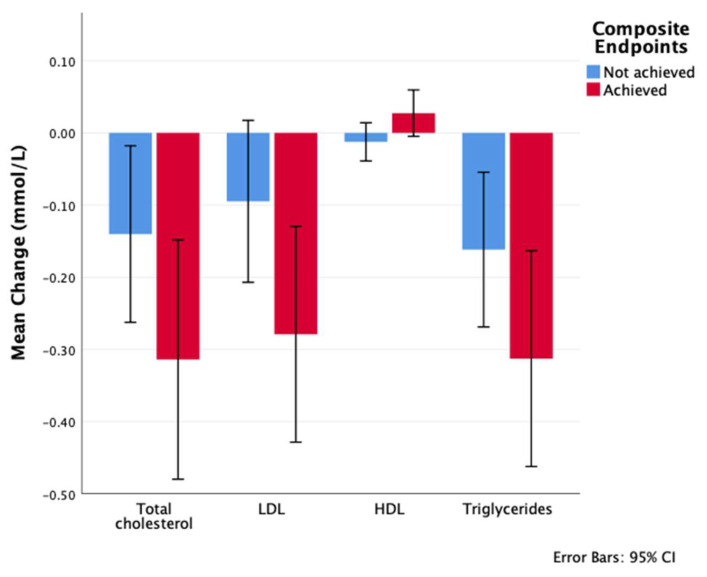
Change in total cholesterol, LDL, HDL, and TG 12 months from baseline by composite endpoint achievement. Refer to Table 3 for the numbers and *p*-values.

Analysis of the changes at 12 months from baseline revealed that the group that achieved the composite endpoint exhibited greater reductions in total cholesterol, LDL, and triglycerides (Table 3). However, the difference in LDL change was the only one that reached statistical significance between the groups. Furthermore, there was a higher increase in HDL levels in the group that achieved the endpoint compared to the group that did not (Figure 6).

### 3.5. Factors Associated with Achieving the Composite Endpoint

Table 4 presents the results of a multivariate logistic regression analysis examining the relationship between the study variables and composite endpoint achievement. The analysis revealed that baseline BMI, baseline A1C, and receiving insulin were significant predictors of composite endpoint achievement, while age, gender, and duration of DM did not predict achievement of the outcome. Higher baseline BMI is not in favor of achieving the composite endpoint (aOR = 0.953; 95% CI: 0.915 to 0.992; *p* = 0.02). Higher baseline A1C is in favor of achieving the composite endpoint (aOR = 1.213; 95% CI: 1.062 to 1.385; *p* = 0.004), and receiving insulin will hinder achievement of the composite endpoint (aOR = 0.02; 95% CI: 0.001 to 0.343; *p* = 0.007). Moreover, the analysis revealed a significant interaction between insulin treatment and baseline BMI (*p* = 0.028). In general, insulin users have a lower rate of achievement, but this is mediated by BMI, indicating that, in patients who are using insulin, those with higher BMI have better achievement of the composite endpoint.

## 4. Discussion

To the best of our knowledge, this is the first real-world study to evaluate the effectiveness of once-weekly semaglutide in achieving a composite endpoint of A1C reduction ≥ 1% and weight loss ≥ 5% in the Saudi population. We found that semaglutide treatment, which lasted for 12 months, was associated with significant reductions in A1C and weight, as well as improvements in the lipid profile. However, less than half of the study cohort (42%) was able to achieve the composite endpoint. The predictors of achieving this endpoint were lower baseline BMI, higher baseline A1C, and insulin non-use.

Data show that nearly a quarter of the Saudi population is recognized as obese, which is one of the highest rates in the world [4]. Furthermore, due to the interrelated aspects of glycemic control and weight management in patients with T2D, we determined a composite endpoint to ensure clinically worthwhile benefits that would be reflected in the prevention of complications.

Obesity is associated with several health conditions, including T2D, hyperlipidemia, cardiovascular disease, hypertension, malignancies, fatty liver, and sleep apnea, as well as an increased risk of mortality [19]. Moreover, weight loss has been found to result in a significant reduction in traditional cardiovascular disease risk factors such as systolic blood pressure, average glucose level, A1C, and triglycerides. The benefits gained from weight loss increase with greater weight reduction, with a linear trend observed [20]. However, improvements in HDL cholesterol and diastolic blood pressure were seen with threshold weight loss of 5 to 10% [21]. In addition to improved glycemic control, weight loss of more than 5% was associated with improved insulin sensitivity and metabolic profile, a decrease in the number of antidiabetic medications used, and an improvement in quality of life [1,20,22]. Adopting a primary weight-centric approach to diabetes treatment would be beneficial for numerous patients with type 2 diabetes [7,23]. Furthermore, weight loss of more than 15% was associated with pronounced metabolic modifications and even remission of type 2 diabetes [23,24]. The benefits of weight loss were evident across all categories of baseline BMI [25].

On the other hand, blood glucose control, as measured by A1C reduction, has been shown to be related to reduced all-cause mortality and microvascular complications and, to a lesser extent, macrovascular complications [26]. Aiming for an A1C level lower than 7% has been shown to have additional benefits on microvascular complications [27,28]. Furthermore, certain medications, such as GLP1-RA and sodium-glucose cotransporter 2 inhibitors, have been found to have a beneficial role in CVD, HF, and CKD, independent of their glucose-lowering effects.

Our findings are consistent with previously reported data on the efficacy of once-weekly semaglutide in significantly reducing both glycemia, as measured by A1C and FBG, and weight after 12 months of treatment with doses of 0.5 mg or 1 mg [9,10,29,30]. Similar to previous studies, we found that patients with higher baseline A1C levels experienced greater reductions in A1C compared to those with lower baseline A1C levels [31]. Correspondingly, we found that semaglutide use was associated with a significant improvement in TC, LDL, and TG levels but not HDL level, irrespective of achieving the composite endpoint. Similar findings have been reported in previous prospective studies [31,32,33]. However, only the degree of LDL reduction was significantly higher in the group that achieved the composite endpoint. This is in general alignment with previous studies [34,35]. Moreover, the HDL level at 12 months was statistically higher in the group that achieved the composite endpoint compared to the group that did not achieve it (1.2 ± 0.29 vs. 1.15 ± 0.27, *p*-value = 0.032). Whether this change is clinically significant is yet to be determined. Previous research showed variant effects on HDL level with semaglutide use [34,36]. High levels of total cholesterol, LDL, and TG and low levels of HDL are well-known cardiovascular risk factors. Therefore, the lowering effect of semaglutide on total cholesterol, LDL, and TG levels, without affecting HDL levels, is in alliance with its cardiovascular protective impact [37].

Our results add to the existing literature real-world experience of achieving a composite endpoint of glycemic and weight improvement in patients with T2D and confirm the findings of previous randomized clinical studies that examined the same composite endpoint. A post hoc analysis of data from SUSTAIN 1 to 5 and 7, which included subjects with T2D who were either drug-naive or on active comparators, showed that 25% to 38% of participants achieved the composite endpoint with 0.5 mg once-weekly semaglutide, while 38% to 59% achieved it with the 1 mg dose [18]. In contrast, our study found that the achievement rates for the composite endpoint were similar for both dosage levels. The absence of a dose-dependent response in our study could be attributed to the relatively short follow-up period or to heterogeneous responses among the participants.

Even though the baseline characteristics were mostly similar between the two groups, multivariate analysis suggests that baseline BMI, baseline A1C, and insulin treatment are important predictors of composite endpoint achievement in the studied population. Having a lower BMI and/or a higher A1C are predictors of achieving the composite endpoint, while insulin use has a disrupting effect on achieving the composite endpoint in general.

The use of exogenous insulin therapy is important in certain T2D patients to gain better glycemic control, yet it is often associated with weight gain [38]. Multiple mechanisms have been proposed to explain this phenomenon, including insulin’s anabolic effect through enhancement of glucose uptake and storage, lipogenesis, and inhibiting protein breakdown. Another mechanism is increasing caloric intake through hunger stimulation and hypoglycemic episodes. Moreover, exogenous insulin promotes fat storage through inhibiting lipolysis and stimulating tissue and fat growth [39,40].

Furthermore, the results of this study suggest that baseline BMI modifies the relationship between insulin use and achievement of the composite endpoint. Specifically, in patients using insulin, each one-unit increase in BMI was associated with approximately 1.09-fold higher odds of achieving the composite outcome. This finding highlights the complex interplay among T2D, obesity, and insulin resistance. While insulin use is typically associated with improved glycemic control, it is also linked to weight gain and worsening insulin resistance, which may limit its overall effectiveness. GLP1-RAs such as semaglutide—promoting both weight loss and glycemic control—may counteract these limitations. Semaglutide may improve insulin sensitivity and enhance the effectiveness of exogenous insulin. Supporting this hypothesis, a previous post hoc analysis of the SUSTAIN 1–3 trials reported that semaglutide reduced insulin resistance primarily through weight loss in patients with type 2 diabetes [41].

Additionally, the doses at 6 and 12 months portrayed a gradual increase for most patients to the recommended maintenance dose of 0.5 to 1 mg once weekly. The majority of patients remained at the same dose throughout the study, and only four patients discontinued treatment mostly for GI side effects. The results from this study show that effectiveness in terms of weight loss and glycemic control was comparable between the two doses. Studies that compared the effectiveness of different doses of semaglutide are minimal. Only one real-world study compared 0.5 and 1 mg in T2D patients and found no difference in effectiveness in terms of weight loss and glycemic control [17]. Another study that looked into the effect of semaglutide on insulin resistance also suggested that the benefit might not be dose-dependent [41]. Further studies are needed to evaluate the effect and clinical benefits of using higher doses of semaglutide.

This study did not evaluate the effect of lifestyle modification, including dietary habits and physical activity, due to its retrospective nature. It is important to note that all patients were offered lifestyle education as part of standard clinical practice, and the results are reflective a real-world scenario. Furthermore, whereas BMI does not differentiate between fat and lean mass, bioelectrical impedance analysis (BIA) provides more accurate information on body composition, including visceral fat percentage, a proven predictor of cardiometabolic risk. However, BIA is not commonly used in clinical practice, making BMI the most practical choice to measure the effect on weight [42].

The current study offers valuable insights into the effectiveness of once-weekly semaglutide, but it is important to acknowledge its limitations. Firstly, the study was conducted at a single center, which could limit the generalizability of our findings to other populations. Secondly, our study design was retrospective, which may be subject to confounding bias and the limitations inherent to the use of medical records. Additionally, the use of a convenience sampling technique may have introduced selection bias into our study population. Overall, while the study has some limitations, we believe that the findings present the outcomes of a clinically important composite endpoint and highlight the need for further research to confirm the appropriate patient profile for this type of treatment, in addition to long-term outcomes.

## 5. Conclusions

Given the close association between T2D and obesity, it is advisable to address these conditions in a combined approach. In addition to its effect on T2D, semaglutide simultaneously addresses various complications. This single-center retrospective study provides further evidence of the remarkable efficacy of once-weekly semaglutide in reducing both weight and A1C levels, as well as improving lipid profiles. Achievement of the composite endpoint of weight loss ≥ 5% and A1C reduction ≥ 1% is highly correlated with clinical outcomes, and the predictors of achieving this endpoint were lower BMI, higher A1C, and insulin non-use. While these findings are promising, further studies are needed to confirm the long-term steady effectiveness of semaglutide and its relation to clinical outcomes.

## Figures and Tables

**Table 1:** Baseline characteristics of total study cohort and subgroups by composite endpoint achievement.

Composite Endpoint	Total	Not Achieved	Achieved	*p* Value
*Number of patients*, n (%)	459	266 (58%)	193 (42%)	
*Age* (years), mean ± SD	52.7 ± 8.5	53.3 ± 8	51.8 ± 9.2	0.053 ^a^
*Diabetes duration* (years)	14.2 ± 7.8	14.6 ± 7.6	13.6 ± 8.1	0.201 ^a^
*Sex/Gender*				
Male	213 (46.4%)	126 (47.4%)	87 (45.1%)	0.637 ^b^
Female	246 (53.6%)	140 (52.6%)	106 (54.9%)	
*Comorbidities*				
Dyslipidemia	417 (90.8%)	241 (90.6%)	176 (91.2%)	0.871 ^b^
Hypertension	247 (53.8%)	143 (53.8%)	104 (53.9%)	1 ^b^
IHD	49 (10.7%)	29 (10.9%)	20 (10.4%)	0.880 ^b^
*Concomitant medications*				
Sulfonylurea	124 (27%)	68 (25.6%)	56 (29%)	0.456 ^b^
Biguanide	450 (98%)	261 (98.1%)	189 (97.9%)	1 ^c^
Glinide	2 (0.4%)	1 (0.4%)	1 (0.5%)	1 ^c^
Thiazolidinedione	5 (1.1%)	4 (1.5%)	1 (0.5%)	0.404 ^c^
SGLT2-I	216 (47.1%)	120 (45.1%)	96 (49.7%)	0.344 ^b^
DPP4-I	14 (3.1%)	9 (3.4%)	5 (2.6%)	0.786 ^c^
Mixed regimen insulin	240 (52.3%)	160 (60.2%)	80 (41.5%)	<0.001 ^b^
Basal only	21 (4.6%)	9 (3.4%)	12 (6.2%)	0.177 ^c^
Bolus only	49 (10.7%)	28 (10.5%)	21 (10.9%)	1 ^c^
*Dose at 6 months*				
0.25 mg	1 (0.2%)	0 (0%)	1 (0.5%)	0.351 ^c^
0.5 mg	199 (43.4%)	120 (45.1%)	79 (40.9%)	
1 mg	259 (56.4%)	146 (54.9%)	113 (58.5%)	
*Dose at 12 months*				
0.25 mg	3 (0.7%)	2 (0.8%)	1 (0.5%)	0.892 ^c^
0.5 mg	106 (23.1%)	63 (23.7%)	43 (22.3%)	
1 mg	350 (76.3%)	201 (75.6%)	149 (77.2%)	

^a^ *p*-value obtained from *t*-test. ^b^ *p*-value obtained from Chi-square test. ^c^ *p*-value obtained from Fisher’s exact test. IHD: Ischemic heart disease. SGLT2-I: Sodium-glucose cotransporter-2 inhibitor. DPP4-I: Dipeptidyl peptidase-4.

**Table 2:** Comparison of baseline characteristics between female and male participants.

	Male	Female	*p* Value
Number of patients, n (%)	213 (46.4%)	246 (53.6%)	
Age (years)	51.7 ± 9.4	53.6 ± 7.6	0.021 ^a^
Diabetes duration (years)	13.6 ± 7.6	14.7 ± 8	0.136 ^a^
Body weight (kg) baseline	96.7 ± 16.5	91.4 ± 14.7	<0.001 ^a^
BMI baseline	34.7 ± 5.3	37.8 ± 5.7	<0.001 ^a^
A1C (%) baseline	9.8 ± 1.5	10.1 ± 1.5	0.041 ^a^
FBG (mmol/L) baseline	11.7 ± 3.9	12 ± 4.1	0.584 ^a^
Total cholesterol (mmol/L) baseline	4.43 ± 1.33	4.54 ± 1.05	0.331 ^a^
HDL (mmol/L) baseline	1.05 ± 0.23	1.27 ± 0.29	0.001 ^a^
LDL (mmol/L) baseline	2.96 ± 1.15	2.92 ± 0.96	0.713 ^a^
TG (mmol/L) baseline	2.11 ± 1.51	1.76 ± 0.82	0.003 ^a^
Comorbidities			
Dyslipidemia	188 (88.3%)	229 (93.1%)	0.077 ^b^
Hypertension	119 (55.9%)	128 (52%)	0.453 ^b^
IHD	31 (14.6%)	18 (7.3%)	0.015 ^b^
Diabetic retinopathy	15 (7%)	19 (7.7%)	0.859 ^b^
Diabetic nephropathy	30 (14.1%)	19 (7.7%)	0.034 ^b^
Concomitant medications			
Sulfonylurea	58 (27.2%)	66 (26.8%)	1 ^b^
Biguanide	208 (97.7%)	242 (98.4%)	0.739 ^c^
Glinide	1 (0.5%)	1 (0.4%)	1 ^c^
Thiazolidinedione	4 (1.9%)	1 (0.4%)	0.188 ^c^
SGLT2 -I	110 (51.6%)	106 (43.1%)	0.075 ^b^
DPP4 inhibitor	5 (2.3%)	9 (3.7%)	0.588 ^c^
Insulin therapy	104 (48.8%)	136 (55.3%)	0.190 ^b^
Sulfonylurea/insulin/thiazolidinedione	170 (79.8%)	221 (89.8%)	0.004 ^b^
Dose at 6 months			
0.25 mg	1 (0.5%)	0 (0%)	0.026 ^c^
0.5 mg	79 (37.1%)	120 (48.8%)	
1 mg	133 (62.4%)	126 (51.2%)	
Dose at 12 months			
0.25 mg	1 (0.5%)	2 (0.8%)	0.866 ^c^
0.5 mg	48 (22.5%)	58 (23.6%)	
1 mg	164 (77%)	186 (75.6%)	

^a^ *p*-value obtained from *t*-test. ^b^ *p*-value obtained from chi-square test. ^c^ *p*-value obtained from Fisher’s exact test. BMI: Body mass index; A1C: hemoglobin A1C; HDL: High-density lipoprotein; LDL: Low-density lipoprotein; IHD: Ischemic heart disease; SGLT2-I: Sodium-glucose cotransporter-2 inhibitor; DPP4-I: Dipeptidyl peptidase-4; TG: Triglycerides.

**Table 3:** Longitudinal changes in metabolic parameters for participants by composite endpoint achievement.

Composite Endpoint	Total	Not Achieved	Achieved	*p* Value
Number of patients, n (%)	459	266 (58%)	193 (42%)	
Composite at 6 months	56 (12.2%)	3 (1.1%)	53 (27.5%)	<0.001 ^b^
Body weight (kg)				
Baseline	93.9 ± 15.8	95.5 ± 15.5	91.7 ± 15.9	0.011 ^a^
6 months	90.6 ± 15.9	93.3 ± 15.5	86.9 ± 15.6	<0.001 ^a^
12 months	87.8 ± 15.9	91.7 ± 15.5	82.5 ± 14.9	<0.001 ^a^
Weight change at 12 months	−6 ± 4.6	−3.7 ± 3.1	−9.1 ± 4.4	<0.001
*p* value of the change from baseline to 12 months ^c^	<0.001	<0.001	<0.001	
BMI				
Baseline	36.3 ± 5.7	36.7 ± 5.7	35.8 ± 5.7	0.111 ^a^
6 months	35.1 ± 5.7	35.9 ± 5.7	34 ± 5.6	0.001 ^a^
12 months	34 ± 5.8	35.2 ± 5.7	32.3 ± 5.5	<0.001 ^a^
BMI change at 12 months	−2.3 ± 1.8	−1.5 ± 1.2	−3.5 ± 1.7	<0.001
*p* value of the change from baseline to 12 months ^c^	<0.001	<0.001	<0.001	
A1C (%)				
Baseline	10 ± 1.5	9.8 ± 1.5	10.2 ± 1.4	0.003 ^a^
6 months	8.5 ± 1.5	8.8 ± 1.4	8.2 ± 1.4	<0.001 ^a^
12 months	7.9 ± 1.5	8.4 ± 1.5	7.2 ± 1.1	<0.001 ^a^
A1c change at 12 months	−2.1 ± 1.8	−1.4 ± 1.6	−3 ± 1.6	<0.001
*p* value of the change from baseline to 12 months ^c^	<0.001	<0.001	<0.001	
FBG (mmol/L)				
Baseline	11.9 ± 4	11.9 ± 4	11.7 ± 3.9	0.611 ^a^
6 months	9.6 ± 3.6	10.1 ± 4	8.9 ± 3	<0.001 ^a^
12 months	8.4 ± 3.3	9.2 ± 3.6	7.4 ± 2.5	<0.001 ^a^
FBG change at 12 months	−3.4 ± 4.3	−2.8 ± 4.2	−4.3 ± 4.2	<0.001
*p* value of the change from baseline to 12 months ^c^	<0.001	<0.001	<0.001	
Total cholesterol (mmol/L)				
Baseline	4.49 ± 1.19	4.42 ± 1.15	4.59 ± 1.23	0.111 ^a^
6 months	4.33 ± 1.08	4.29 ± 1.09	4.4 ± 1.08	0.295 ^a^
12 months	4.27 ± 1.08	4.28 ± 1.11	4.27 ± 1.05	0.967 ^a^
Cholesterol change at 12 months	−0.2 ± 1.1	−0.1 ± 1	−0.3 ± 1.2	0.090
*p* value of the change from baseline to 12 months ^c^	<0.001	0.025	<0.001	
HDL (mmol/L)				
Baseline	1.17 ± 0.29	1.16 ± 0.29	1.18 ± 0.28	0.558 ^a^
6 months	1.16 ± 0.29	1.14 ± 0.3	1.19 ± 0.26	0.092 ^a^
12 months	1.17 ± 0.28	1.15 ± 0.27	1.2 ± 0.29	0.032 ^a^
HDL change at 12 months	0 ± 0.7	−0.1 ± 1	0 ± 0.2	0.421
*p* value of the change from baseline to 12 months ^c^	0.803	0.360	0.093	
LDL (mmol/L)				
Baseline	2.94 ± 1.05	2.88 ± 1.03	3.02 ± 1.08	0.147 ^a^
6 months	2.82 ± 0.97	2.79 ± 0.97	2.86 ± 0.98	0.450 ^a^
12 months	2.76 ± 0.97	2.78 ± 0.99	2.73 ± 0.94	0.585 ^a^
LDL change at 12 months	−0.2 ± 1	−0.1 ± 0.9	−0.3 ± 1.1	0.048
*p* value of the change from baseline to 12 months ^c^	<0.001	0.098	<0.001	
TG (mmol/L)				
Baseline	1.92 ± 1.2	1.89 ± 1.01	1.98 ± 1.43	0.409 ^a^
6 months	1.75 ± 0.79	1.76 ± 0.81	1.73 ± 0.77	0.608 ^a^
12 months	1.7 ± 0.88	1.72 ± 0.86	1.67 ± 0.91	0.486 ^a^
TG change at 12 months	−0.2 ± 1	−0.2 ± 0.9	−0.3 ± 1.1	0.097 ^a^
*p* value of the change from baseline to 12 months ^c^	<0.001	0.003	<0.001	

^a^ *p*-value obtained from *t*-test. ^b^ *p*-value obtained from Fisher’s exact test. ^c^ *p*-value obtained from paired *t*-test. BMI: Body mass index; A1C: Hemoglobin A1C; FBG: Fasting blood glucose; HDL: High-density lipoprotein; LDL: Low-density lipoprotein; IHD: Ischemic heart disease; SGLT2-I: Sodium-glucose cotransporter-2 inhibitor; DPP4-I: Dipeptidyl peptidase-4; TG: Triglycerides.

**Table 4:** Multivariate logistic regression analysis for predictors of composite endpoint achievement.

	aOR ^†^	95% CI	*p* Value
Age (year)	0.983	0.958 to 1.009	0.189
Male	reference		
Female	1.273	0.846 to 1.915	0.247
Duration of T2D	1.006	0.978 to 1.035	0.686
Baseline BMI	0.953	0.915 to 0.992	0.02
Baseline A1C	1.213	1.062 to 1.385	0.004
Insulin	0.02	0.001 to 0.343	0.007
Insulin * baseline BMI	1.092	1.009 to 1.181	0.028

^†^ Adjusted odds ratio. BMI: Body mass index; T2D: Type 2 diabetes mellitus; A1C: Hemoglobin A1C.

## Data Availability

The data that support the findings of this study are available from the corresponding author upon reasonable request.

## Abbreviations

BMI	Body mass index
A1C	Hemoglobin A1C
CI	Confidence interval
CVD	Cardiovascular disease
IHD	Ischemic heart disease
eGFR	Estimated glomerular filtration rate
FBG	Fasting blood glucose
GLP1-RA	Glucagon-like peptide-1 receptor agonist
HDL	High-density lipoprotein
LDL	Low-density lipoprotein
SGLT2-I	Sodium-glucose cotransporter-2 inhibitors
T2D	Type 2 diabetes mellitus
TG	Triglycerides
DPP4-I	Dipeptidyl peptidase-4

## References

[1] Aras M., Tchang B.G., Pape J. (2021). Obesity and Diabetes. Nurs. Clin. N. Am..

[2] Boden G. (2011). Obesity, insulin resistance and free fatty acids. Curr. Opin. Endocrinol. Diabetes Obes..

[3] Katsiki N., Anagnostis P., Kotsa K., Goulis D.G., Mikhailidis D.P. (2019). Obesity, Metabolic Syndrome and the Risk of Microvascular Complications in Patients with Diabetes mellitus. Curr. Pharm. Des..

[4] Althumiri N.A., Basyouni M.H., AlMousa N., AlJuwaysim M.F., Almubark R.A., BinDhim N.F., Alkhamaali Z., Alqahtani S.A. (2021). Obesity in Saudi Arabia in 2020: Prevalence, Distribution, and Its Current Association with Various Health Conditions. Healthcare.

[5] Chong B., Jayabaskaran J., Kong G., Chan Y.H., Chin Y.H., Goh R., Kannan S., Ng C.H., Loong S., Kueh M.T.W. (2023). Trends and predictions of malnutrition and obesity in 204 countries and territories: An analysis of the Global Burden of Disease Study 2019. eClinicalMedicine.

[6] Ammori B.J., Skarulis M.C., Soran H., Syed A.A., Eledrisi M., Malik R.A. (2020). Medical and surgical management of obesity and diabetes: What’s new?. Diabet. Med..

[7] Davies M.J., Aroda V.R., Collins B.S., Gabbay R.A., Green J., Maruthur N.M., Rosas S.E., Del Prato S., Mathieu C., Mingrone G. (2022). Management of Hyperglycemia in Type 2 Diabetes, 2022. A Consensus Report by the American Diabetes Association (ADA) and the European Association for the Study of Diabetes (EASD). Diabetes Care.

[8] Apovian C.M., Okemah J., O’Neil P.M. (2019). Body Weight Considerations in the Management of Type 2 Diabetes. Adv. Ther..

[9] Hall S., Isaacs D., Clements J.N. (2018). Pharmacokinetics and Clinical Implications of Semaglutide: A New Glucagon-Like Peptide (GLP)-1 Receptor Agonist. Clin. Pharmacokinet..

[10] Sorli C., Harashima S.I., Tsoukas G.M., Unger J., Karsbøl J.D., Hansen T., Bain S.C. (2017). Efficacy and safety of once-weekly semaglutide monotherapy versus placebo in patients with type 2 diabetes (SUSTAIN 1): A double-blind, randomised, placebo-controlled, parallel-group, multinational, multicentre phase 3a trial. Lancet Diabetes Endocrinol..

[11] Kaneko M., Narukawa M. (2018). Assessment of Cardiovascular Risk With Glucagon-Like Peptide 1 Receptor Agonists in Patients With Type 2 Diabetes Using an Alternative Measure to the Hazard Ratio. Ann. Pharmacother..

[12] Smits M.M., Van Raalte D.H. (2021). Safety of Semaglutide. Front. Endocrinol..

[13] Al Hayek A.A., Al Dawish M.A. (2022). Evaluation of Patient-Reported Satisfaction and Clinical Efficacy of Once-Weekly Semaglutide in Patients with Type 2 Diabetes: An Ambispective Study. Adv. Ther..

[14] Einarson T.R., Garg M., Kaur V., Hemels M.E.H. (2014). Composite endpoints in trials of type-2 diabetes. Diabetes Obes. Metab..

[15] Yale J.F., Bodholdt U., Catarig A.M., Catrina S., Clark A., Ekberg N.R., Erhan U., Holmes P., Knudsen S.T., Liutkus J. (2022). Real-world use of once-weekly semaglutide in patients with type 2 diabetes: Pooled analysis of data from four SURE studies by baseline characteristic subgroups. BMJ Open Diabetes Res Care..

[16] Dungan K.M., Bardtrum L., Christiansen E., Eliasson J., Mellbin L., Woo V.C., Vilsbøll T. (2023). Greater Combined Reductions of HbA1c ≥ 1.0% and Body Weight Loss ≥ 5.0% or ≥10.0% with Orally Administered Semaglutide Versus Comparators. Diabetes Ther..

[17] Alenzi S., Alzahrani A., Aljaloud A., Alanazi K., Alarfaj S.J. (2024). The effectiveness of 0.5 mg and 1 mg of semaglutide in patients with type two diabetes and predictors of response: A retrospective cohort study. Front. Endocrinol..

[18] Rodbard H.W., Bellary S., Hramiak I., Seino Y., Silver R., Damgaard L.H., Nayak G., Zacho J., Aroda V.R. (2019). Greater Combined Reductions in HbA1c ≥1.0% and Weight ≥5.0% with Semaglutide versus Comparators In Type 2 Diabetes. Endocr. Pr..

[19] Calle E.E., Thun M.J., Petrelli J.M., Rodriguez C., Heath C.W. (1999). Body-mass index and mortality in a prospective cohort of U.S. adults. N. Engl. J. Med..

[20] Wing R.R., Lang W., Wadden T.A., Safford M., Knowler W.C., Bertoni A.G., Hill J.O., Brancati F.L., Peters A., Wagenknecht L. (2011). Benefits of modest weight loss in improving cardiovascular risk factors in overweight and obese individuals with type 2 diabetes. Diabetes Care.

[21] Ryan D.H., Yockey S.R. (2017). Weight Loss and Improvement in Comorbidity: Differences at 5%, 10%, 15%, and Over. Curr. Obes. Rep..

[22] Magkos F., Fraterrigo G., Yoshino J., Luecking C., Kirbach K., Kelly S.C., de Las Fuentes L., He S., Okunade A.L., Patterson B.W. (2016). Effects of Moderate and Subsequent Progressive Weight Loss on Metabolic Function and Adipose Tissue Biology in Humans with Obesity. Cell Metab..

[23] Lingvay I., Sumithran P., Cohen R.V., le Roux C.W. (2022). Obesity management as a primary treatment goal for type 2 diabetes: Time to reframe the conversation. Lancet.

[24] Lean M.E., Leslie W.S., Barnes A.C., Brosnahan N., Thom G., McCombie L., Peters C., Zhyzhneuskaya S., Al-Mrabeh A., Hollingsworth K.G. (2018). Primary care-led weight management for remission of type 2 diabetes (DiRECT): An open-label, cluster-randomised trial. Lancet.

[25] Unick J.L., Beavers D., Jakicic J.M., Kitabchi A.E., Knowler W.C., Wadden T.A., Wing R.R. (2011). Effectiveness of lifestyle interventions for individuals with severe obesity and type 2 diabetes: Results from the Look AHEAD trial. Diabetes Care.

[26] Lind M., Imberg H., Coleman R.L., Nerman O., Holman R.R. (2021). Historical HbA1c Values May Explain the Type 2 Diabetes Legacy Effect: UKPDS 88. Diabetes Care.

[27] Sun S., Hisland L., Grenet G., Gueyffier F., Cornu C., Jaafari N., Boussageon R. (2022). Reappraisal of the efficacy of intensive glycaemic control on microvascular complications in patients with type 2 diabetes: A meta-analysis of randomised control-trials. Therapies.

[28] Agrawal L., Azad N., Bahn G.D., Ge L., Reaven P.D., Hayward R.A., Reda D.J., Emanuele N.V., VADT Study Group (2018). Long-term follow-up of intensive glycaemic control on renal outcomes in the Veterans Affairs Diabetes Trial (VADT). Diabetologia.

[29] Zaazouee M.S., Hamdallah A., Helmy S.K., Hasabo E.A., Sayed A.K., Gbreel M.I., Elmegeed A.A., Aladwan H., Elshanbary A.A., Abdel-Aziz W. (2022). Semaglutide for the treatment of type 2 Diabetes Mellitus: A systematic review and network meta-analysis of safety and efficacy outcomes. Diabetes Metab. Syndr..

[30] Shi F.H., Li H., Cui M., Zhang Z.L., Gu Z.C., Liu X.Y. (2018). Efficacy and Safety of Once-Weekly Semaglutide for the Treatment of Type 2 Diabetes: A Systematic Review and Meta-Analysis of Randomized Controlled Trials. Front. Pharmacol..

[31] Okamoto A., Yokokawa H., Nagamine T., Fukuda H., Hisaoka T., Naito T. (2021). Efficacy and safety of semaglutide in glycemic control, body weight management, lipid profiles and other biomarkers among obese type 2 diabetes patients initiated or switched to semaglutide from other GLP-1 receptor agonists. J. Diabetes Metab. Disord..

[32] Aroda V.R., Ahmann A., Cariou B., Chow F., Davies M.J., Jódar E., Mehta R., Woo V., Lingvay I. (2019). Comparative efficacy, safety, and cardiovascular outcomes with once-weekly subcutaneous semaglutide in the treatment of type 2 diabetes: Insights from the SUSTAIN 1-7 trials. Diabetes Metab..

[33] Yamada H., Yoshida M., Funazaki S., Morimoto J., Tonezawa S., Takahashi A., Nagashima S., Masahiko K., Kiyoshi O., Hara K. (2023). Retrospective Analysis of the Effectiveness of Oral Semaglutide in Type 2 Diabetes Mellitus and Its Effect on Cardiometabolic Parameters in Japanese Clinical Settings. J. Cardiovasc. Dev. Dis..

[34] Di Loreto C., Minarelli V., Nasini G., Norgiolini R., Del Sindaco P. (2022). Effectiveness in Real World of Once Weekly Semaglutide in People with Type 2 Diabetes: Glucagon-Like Peptide Receptor Agonist Naïve or Switchers from Other Glucagon-Like Peptide Receptor Agonists: Results from a Retrospective Observational Study in Umbria. Diabetes Ther..

[35] Pérez-Belmonte L.M., Sanz-Cánovas J., García de Lucas M.D., Ricci M., Avilés-Bueno B., Cobos-Palacios L., Pérez-Velasco M.A., López-Sampalo A., Bernal-López M.R., Jansen-Chaparro S. (2022). Efficacy and Safety of Semaglutide for the Management of Obese Patients With Type 2 Diabetes and Chronic Heart Failure in Real-World Clinical Practice. Front. Endocrinol..

[36] Masaki T., Ozeki Y., Yoshida Y., Okamoto M., Miyamoto S., Gotoh K., Shibata H. (2022). Glucagon-Like Peptide-1 Receptor Agonist Semaglutide Improves Eating Behavior and Glycemic Control in Japanese Obese Type 2 Diabetic Patients. Metabolites.

[37] Goldberg R.B. (2000). Hyperlipidemia and cardiovascular risk factors in patients with type 2 diabetes. Am. J. Manag. Care.

[38] Lee S.H., Park S.Y., Choi C.S. (2022). Insulin Resistance: From Mechanisms to Therapeutic Strategies. Diabetes Metab. J..

[39] Russell-Jones D., Khan R. (2007). Insulin-associated weight gain in diabetes -causes, effects and coping strategies. Diabetes Obes. Metab..

[40] Anekwe C.V., Ahn Y.J., Bajaj S.S., Stanford F.C. (2024). Pharmacotherapy causing weight gain and metabolic alteration in those with obesity and obesity-related conditions: A review. Ann. N. Y. Acad. Sci..

[41] Fonseca V.A., Capehorn M.S., Garg S.K., Gimeno E.J., Hansen O.H., Holst A.G., Nayak G., Seufert J. (2019). Reductions in Insulin Resistance are Mediated Primarily via Weight Loss in Subjects with Type 2 Diabetes on Semaglutide. J. Clin. Endocrinol. Metab..

[42] Wu Y., Li D., Vermund S.H. (2024). Advantages and Limitations of the Body Mass Index (BMI) to Assess Adult Obesity. Int. J. Environ. Res. Public Health.

